# Internal 3D temperature mapping in biological systems using ratiometric light-sheet imaging and lipid-coated upconversion nanothermometers

**DOI:** 10.3762/bjnano.16.159

**Published:** 2025-12-22

**Authors:** Dannareli Barron-Ortiz, Enric Pérez-Parets, Rubén D Cadena-Nava, Emilio J Gualda, Jacob Licea-Rodríguez, Juan Hernández-Cordero, Pablo Loza-Álvarez, Israel Rocha-Mendoza

**Affiliations:** 1 Centro de Investigación Científica y de Educación Superior de Ensenada (CICESE), Carretera Ensenada-Tijuana, No. 3918, Zona Playitas, Ensenada 22860, México; 2 ICFO-Institut de Ciencies Fotoniques, The Barcelona Institute of Science and Technology, Av. Carl Friedrich Gauss, 3, 08860 Castelldefels, Spain; 3 Centro de Nanociencias y Nanotecnología (CNyN), Universidad Nacional Autónoma de México (UNAM), Km 107 Carretera Tijuana-Ensenada, Pedregal Playitas, Ensenada 22860, México; 4 Department of Agri-Food Engineering and Biotechnology (DEAB), Universitat Politècnica de Catalunya, Esteve Terradas 8, 08860 Castelldefels, Spain; 5 Centro de Investigación en Ingeniería y Ciencias Aplicadas (CIICAp), Universidad Autónoma del Estado de Morelos, Cuernavaca 62209, México; 6 Instituto de Investigaciones en Materiales, Universidad Nacional Autónoma de México, A.P. 70-360, México City 04510, México

**Keywords:** *C. elegans*, 3D imaging, fluorescent intensity ratio, light-sheet microscopy, temperature mapping, upconversion fluorescent nanoparticles

## Abstract

Upconversion nanoparticles (UCNPs) are well-known for their high efficiency, photostability, near-infrared excitation, and ability to estimate temperature through ratiometric imaging of two thermally coupled fluorescence bands. This work demonstrates the feasibility of volumetric temperature mapping in internal biological systems using light-sheet fluorescence microscopy and lipid-coated UCNPs as nanothermometry markers. This approach enables real-time thermal mapping with both high spatial and temporal resolution at the cellular and subcellular levels. To validate the method, we performed 3D temperature imaging on fixed *Caenorhabditis elegans* (*C. elegans*) after UCNP ingestion. The proposed technique represents a cutting-edge method for accurate 3D analysis of temperature-driven biological processes. It holds significant potential for applications in living organisms, offering a non-invasive tool to monitor intracellular and organ-specific temperature dynamics.

## Introduction

Biological processes involving energy exchange often manifest as temperature fluctuations. Materials sought to measure such changes should exhibit high sensitivity, accuracy, high spatiotemporal resolution, good biocompatibility, low cytotoxicity, and stable optical and chemical properties. Additionally, given the conditions commonly presented in biological samples, these materials should also remain unaffected by changes in pH, concentration, ionic strength, and viscosity [1]. Traditional thermometers are macroscopic devices with several disadvantages, including limited sensitivity and low accuracy, and are generally restricted to contact surface measurements [2]. Beyond fundamental processes, temperature also serves as a key biomarker for pathological conditions such as cancer. Localized hyperthermia often arises from dysregulated metabolism (i.e., the Warburg effect) [3–4] and chaotic vasculature that impairs heat dissipation [5–6]. These factors can create thermal gradients of 0.5–2.0 °C between tumors and healthy tissue, with even greater differences at the subcellular level [7–9]. Consequently, the ability to map temperature with high spatial resolution is a critical tool for probing disease mechanisms, potentially aiding in diagnostics and therapeutic evaluation. To address these limitations, luminescent nanomaterial-based thermometers (LNTs) have emerged as promising alternatives for biological and non-biological applications. LNTs rely on the emission properties of a fluorophore and its thermal dependence to measure temperature changes, which can be measured as variations in the emission intensity [10–13] and lifetime [8,14–16], spectral shift [17], as well as intensity ratios [18–29] and polarization anisotropy [30–31].

In biological applications involving fluorescent nanothermometry, light–tissue interactions must be carefully considered for an accurate temperature measurement. To address this, a wide range of luminescent materials have been developed, including nanodiamonds [32], quantum dots [17,28], nanodots [11,13,33], fluorescent-based molecular systems [1,8,34], and lanthanide (Ln^3+^)-doped materials [10,35–37]. Among all these alternatives, lanthanide-doped materials offer a distinct advantage: upconversion (UC) fluorescence, enabling the conversion of low-energy excitation (longer wavelengths) into high-energy emission (shorter wavelengths). This is particularly advantageous for biomedical applications, as it eliminates the need for ultraviolet or visible excitation, which can cause photobleaching and phototoxicity [38]. The ladder-like energy level structure of Ln^3+^ ions enable efficient photon UC of near-infrared (NIR) light, even with moderate excitation intensities (1–10^3^ W·cm^−2^) attainable with gas-based lamps or continuous wave lasers [39].

**Figure 1:**
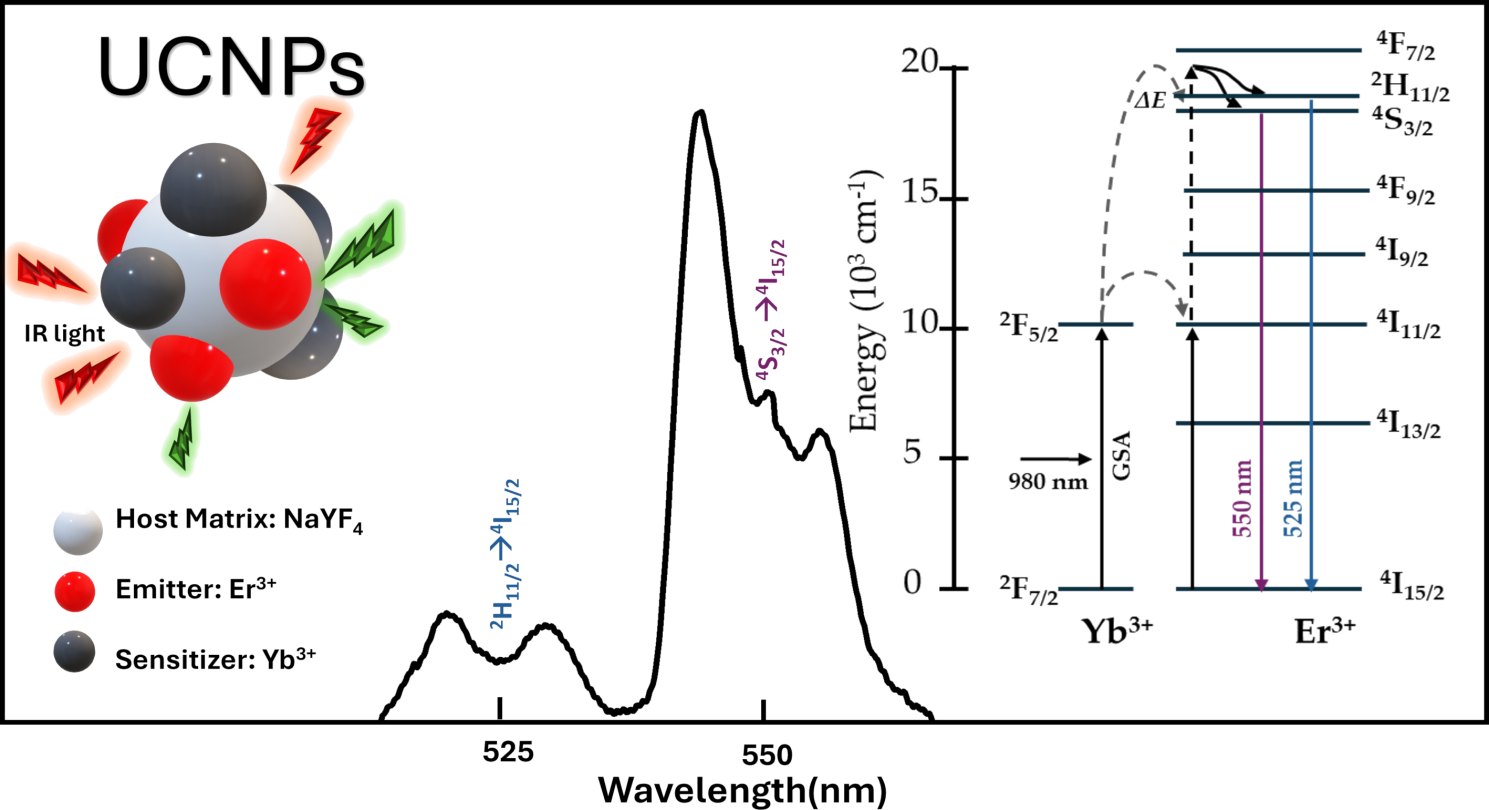
Characteristic UC fluorescent emission spectra of NaYF_4_:Er^3+^/Yb^3+^ in the green region when irradiated with 980 nm laser light. Insets: UC process scheme (right). Schematic representation of the energy transfer between Yb^3+^ and Er^3+^ ions and the UC emission in an upconversion nanoparticle UCNP (left).

Temperature measurements using Ln^3+^-doped nanomaterials have mainly focused on using UC nanoparticles (UCNPs), which are inorganic crystalline structures, typically composed of sodium yttrium fluoride (NaYF_4_) co-doped with rare-earth (RE) ions like ytterbium (Yb^3+^), erbium (Er^3+^), and gadolinium (Gd^3+^). These RE ions act as sensitizers and emitters, allowing for photonic UC of multiple NIR photons into visible luminescence [38]. As an example, in the NaYF_4_:Er^3+^/Yb^3+^ composite (such as that used in this work), the NaYF_4_ crystal matrix is co-doped with Yb^3+^ acting as the sensitizer to enhance the NIR absorption cross section, whereas the Er^3+^ ion acts as the emitter, as depicted in Figure 1.

When UCNPs of this composite are irradiated with 980 nm NIR light, they emit two thermally coupled green fluorescence bands at 525 and 550 nm. These bands arise from the ^2^H_11/2_→^4^I_15/2_ (blue) and ^4^S_3/2_→^4^I_15/2_ (purple) transitions of Er^3+^ ions, respectively [39–41]. Thermally coupled bands, defined as bands separated by less than 2000 cm^−1^ (<0.248 eV), favor a higher-level population with an increase in thermal energy, thereby enabling temperature-sensitive fluorescence emission [42]. For the NaYF_4_:Er^3+^/Yb^3+^ composite, the energy bands (^2^H_11/2_ and ^4^S_3/2_) are separated by approximately 866 cm^−1^; they are thus thermally coupled, and the ratio of their intensities provides a reliable means for temperature monitoring. This approach is known as the fluorescence intensity ratio (FIR), described mathematically as:


[1] $$\text{FIR}=\frac{I_{525}}{I_{550}}=C\exp\left(\frac{-\Delta E}{kT}\right),$$

where *I*_525_ and *I*_550_ are the integrated intensities of the two fluorescence bands centered at 525 and 550 nm, respectively; ∆*E* is the energy difference between the ^2^H_11/2_ and ^4^S_4/2_ levels, *k* is Boltzmann’s constant (0.695 cm^−1^·K^−1^), *T* is the absolute temperature in Kelvin (K), and *C* is a constant associated with the host material and determined by the degeneracy of the coupled energy levels, emission frequencies, and spontaneous radiation transition rates [43].

The FIR-based technique has been widely used for optical thermometry given its inherent advantages, including noise cancellation capabilities, real-time temperature sensing, and high sensitivity [44]. These features make FIR-based thermometry appealing for remote optical measurements in biological applications. Indeed, upon imaging the thermally coupled fluorescent bands emitted by UCNPs with microscopy techniques, precise temperature measurements within biological systems can be readily obtained. For instance, Vetrone et al. [36] used UCNP@PEI as nanothermometers for two-dimensional (2D) temperature mapping inside Hella cells. Similarly, Piñol and co-workers [20] used the FIR of Ln^3+^ (Sm, Eu)-bearing polymeric micelles to achieve real-time 2D temperature maps of breast metastatic adenocarcinoma cells. Di et al. [45] used functionalized UCNPs to monitor mitochondrial thermal dynamics in HeLa cells, and Wang et al. [37] used core–shell UCNPs to monitor temperature and imaging inside and outside onion cells simultaneously. These studies underscore the versatility and potential of UCNP-based thermometry for non-invasive, precise temperature measurements and cell imaging.

Despite these advancements, most approaches rely on 2D imaging or point-scanning spectroscopic methods to generate temperature maps, which are limited in axial resolution and acquisition speed when applied to thick or heterogeneous biological samples. Earlier studies using luminescent nanothermometers have demonstrated spatially resolved, yet essentially planar, temperature mapping in transparent or thin systems [24–25]. More recently, efforts in luminescence-based 3D thermometry at the cellular scale [46–47] have explored volumetric temperature sensing through fluorescence-lifetime or UC nanothermometry. However, these approaches remain limited by point-by-point scanning, shallow penetration depth, and narrow fields of view (typically below 100 µm), restricting their applicability to intact organisms. Beyond these demonstrations, recent reviews [48–49] emphasize that achieving true 3D luminescence thermometry remains a major challenge as most implementations rely on complex instrumentation and confined imaging volumes. Therefore, extending nanothermometry toward fast, high-resolution volumetric mapping represents an essential step for advancing non-invasive thermal imaging in living biological systems.

We recently demonstrated the feasibility of combining light-sheet fluorescence microscopy (LSFM) with UC micro- and nanocomposites for volumetric temperature mapping across scales ranging from tens of micrometers to millimeters [40]. LSFM decouples excitation and detection, illuminating only the focal plane and thereby minimizing photobleaching and photothermal effects while enabling rapid volumetric acquisition. The present work aims to extend this technique to larger biological specimens, specifically *C. elegans*, an optically transparent model organism ideally suited for LSFM due to its simple “tube-within-a-tube” anatomy and clearly defined internal organs. When the nematode is fed with lipid-coated UCNPs, the nanoparticles localize within its digestive tract, acting as thermosensitive markers and enabling voxel-resolved, real-time 3D thermal mapping with exceptional spatial and temporal resolution [45,50–51], revealing internal temperature gradients inaccessible with 2D imaging. The results reported herein highlight the potential of LSFM and FIR-based thermometry as a non-invasive method for precise temperature mapping in living organisms.

## Materials and Methods

### Lipid-wrapped UCNPs

**Figure 2:**
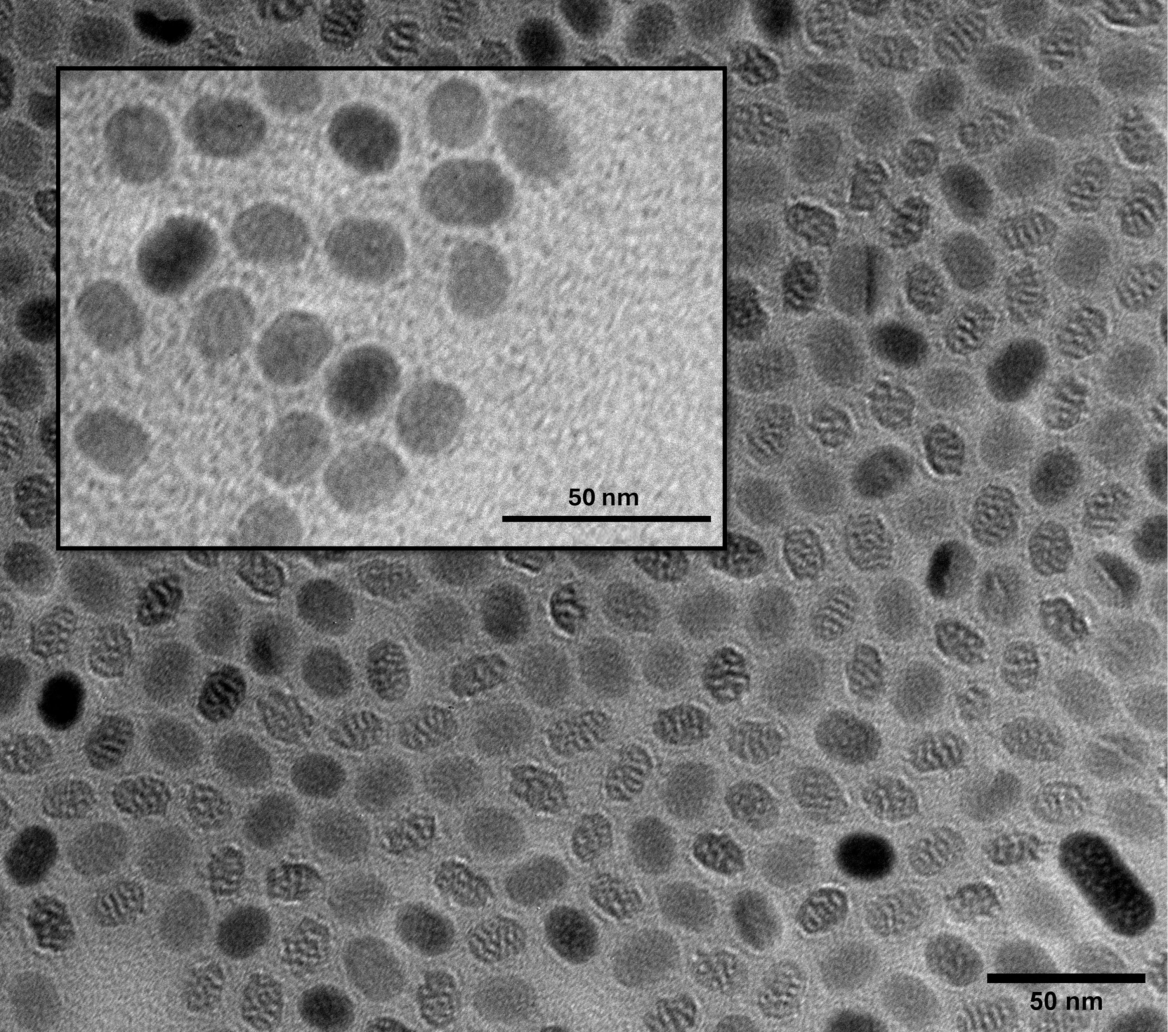
TEM micrograph of as-bought UCNPs dispersed in toluene. Inset: functionalized UCNPs@lipids dispersed in Milli-Q water. Scale bar: 50 nm.

Due to their highly hydrophobic nature, commercial NaYF_4_:Yb^3+^/Er^3+^ UCNPs (Sigma-Aldrich, No. 900556 1 ML) were coated with lipids to enhance their water dispersibility. This lipid coating (UCNPs@lipids) was applied using a modified thin-film hydration method based on Rojas-Gutierrez’s procedure [52]. Based on the size distribution and concentration of the nanoparticles (10 mg·mL^−1^), a lipid layer was formed with DOPS, cholesterol, and DMPC at a molar ratio of 64:29:7. The three lipids were dispersed in chloroform and mixed with 100 µL of UCNPs in a round-bottomed flask. The resulting mixture was then evaporated under a constant flow of N_2_ gas while stirring in a circular motion for 30 min. After solvent evaporation, 2 mL of Milli-Q water were added to the flask for rehydration overnight at 4 °C. The resulting water-dispersible UCNPs@lipids solution had a UCNP concentration of 1 mg·mL^−1^ and was stored at room temperature for subsequent use and characterization. Evidence of obtaining such a water-dispersible solution was previously reported in [40]. Figure 2 compares the as-purchased nanoparticles dispersed in toluene with the lipid-functionalized UCNPs (inset), revealing a size distribution of approximately 15–20 nm. Because the lipid shell is an ultrathin organic layer (2–3 nm) with low electron contrast, no distinct morphological differences are expected between coated and uncoated nanoparticles in TEM images; however, the success of the coating is confirmed by the stable dispersion of UCNPs in water, which prevents aggregation.

### *C. elegans* culture and maintenance

The nematodes used in our experiments*,* N2 wild-type *C. elegans,* were cultivated on nematode growth medium (NGM) plates previously seeded with *E. coli* OP50. Nematode transfer was performed every two days, moving a chunk of agar from a three-to-five-day-old plate to a new NGM plate seeded with *E. coli*. The nematodes were cultivated at room temperature (≈23 °C).

#### *C. elegans* feeding with UCNPs

To feed the nematodes with UCNPs, ten to twelve nematodes, each approximately 1 mm in length, were selected and individually transferred from a three-day-old cultivation plate to a fresh small NGM plate (35 mm in diameter) that was not seeded with *E. coli* OP50. The nematodes transfer was gently conducted using a stereomicroscope (ZEISS, Stemi 2000) equipped with a transmitted light source and thin tweezers. After the transfer, 50 µL of the UCNPs@lipids solution was carefully dropped onto the plate and spread to cover most of the plate. Before imaging, the nematodes were left in contact with the UCNPs@lipids solutions for approximately 17 h.

#### Samples used for temperature mapping

**Figure 3:**
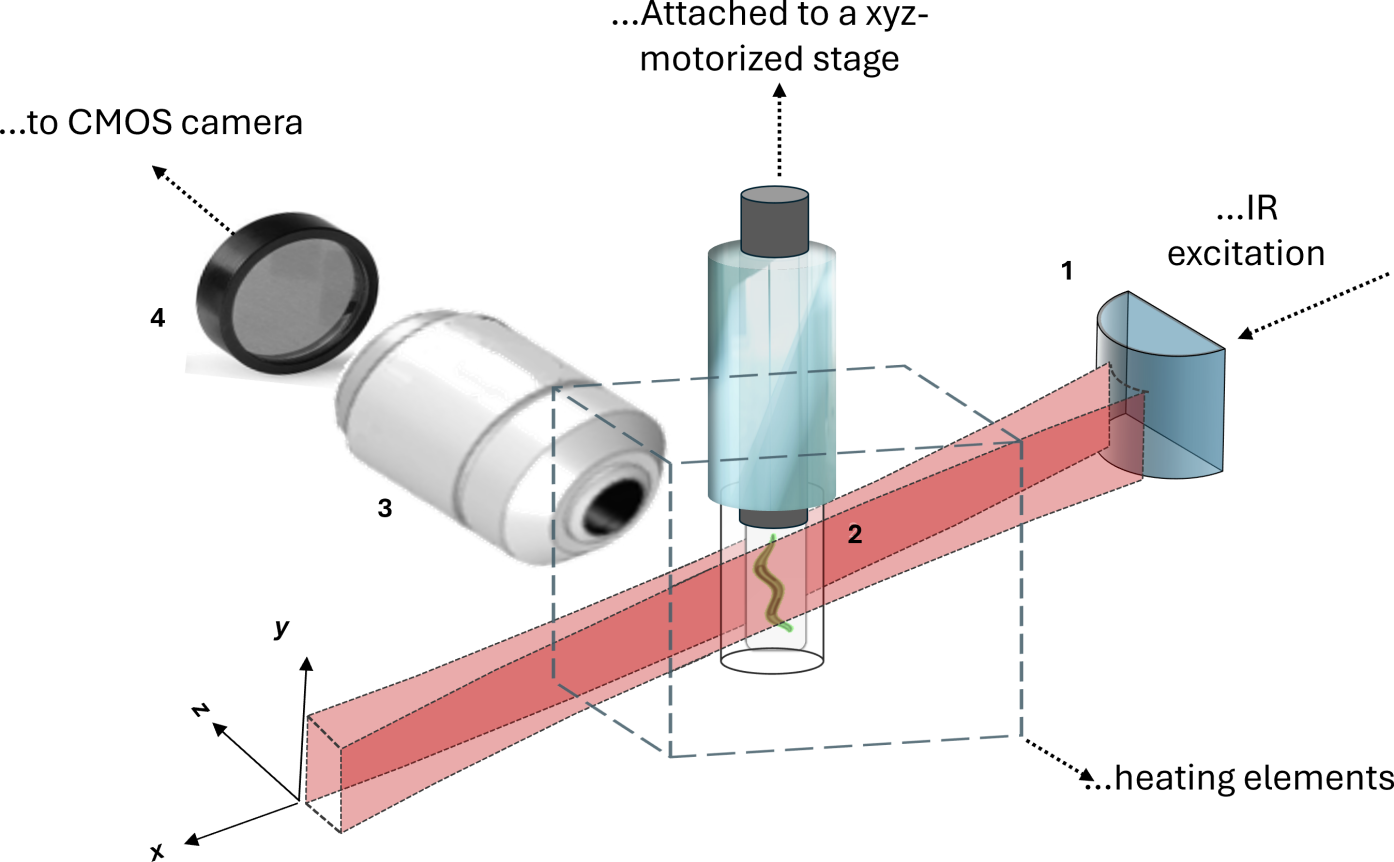
General scheme of the sample mounting holder used for temperature mapping in the light sheet microscopy system. The numbers in the figure stand for cylindrical optics (1), sample (2), collection objective (3), and optical filter set (4). Figure 3 was adapted from [40] (© 2023 D. Barron-Ortiz et al., published by MDPI, distributed under the terms of the Creative Commons Attribution 4.0 International License, https://creativecommons.org/licenses/by/4.0).

Two types of samples were prepared in our experiments, one for FIR-temperature calibration and the other for internal temperature mapping in nematodes. The first sample, S1, was an agarose phantom with lipid-coated UCNPs, as reported in [40]. It consisted of a mixture of 250 µL of UCNPs@lipids solution and 250 µL of 2% low-melting agarose gel. A few microliters of the liquid mixture were drawn into a fluorinated ethylene propylene (FEP) tube and solidified 30 min before imaging. The second sample, S2, consisted of a single UC-fed *C. elegans* immersed in agarose mixed with UCNPs@lipids. After feeding with UCNPs@lipids, live nematodes were placed into a drop of 2% low-melting agarose gel containing UCNPs@lipids and then fixed with 4% paraformaldehyde (PFA). The nematodes were oriented with their mouth and tail positioned at the ends of the FEP tube and left to solidify for 30 min before imaging. The FEP tubes were inserted into a capillary tube for structural support to mount the samples. Using a metal plunger, the samples were drawn into the exposed end of the FEP tube, as illustrated in Figure 3.

#### Light sheet microscopy

The LSFM setup is described in [40]. Here, the NIR excitation at 980 nm was provided by a fiber Bragg grating-stabilized laser (JDSU, 2900 Series), with collimated light focused onto the back focal plane of a 4×/0.13 NA excitation objective (Nikon, PlanFluor) using a 250 mm cylindrical lens (Thorlabs, LJ1267RM-A). The excitation objective generated the light sheet in the sample’s *xy*-plane, with a capillary glass mounted on an *xyz*-motorized stage (Thorlabs, 3-axis NanoMax) for depth imaging. Images were captured using a CMOS camera (Hamamatsu, ORCA-Flash4.0) with a 10×/0.3 NA water immersion objective (Nikon, PlanFluor) and tube lens, achieving a 1.3 × 1.3 mm^2^ FOV and ≈1.3 μm axial resolution. Two interferometric filters (Thorlabs, MF510-40, and MF559-34) isolated the 525 and 550 nm UC fluorescence bands, while a bandpass filter (Thorlabs, FESH-0650) blocked the excitation light.

To heat the immersed sample, a custom-made immersion chamber was used to house the capillary tube. The chamber featured a temperature-controlled water recirculation system consisting of a peristaltic pump (KF Technology, NE-9000), a solution heater (Warner Instruments, Hamden), and a temperature controller (Warner Instruments, TC-324C). This setup enabled precise control from 25 to 50 °C with ±0.2 °C accuracy.

## Results and Discussion

### Three-dimensional calibration curves

**Figure 4:**
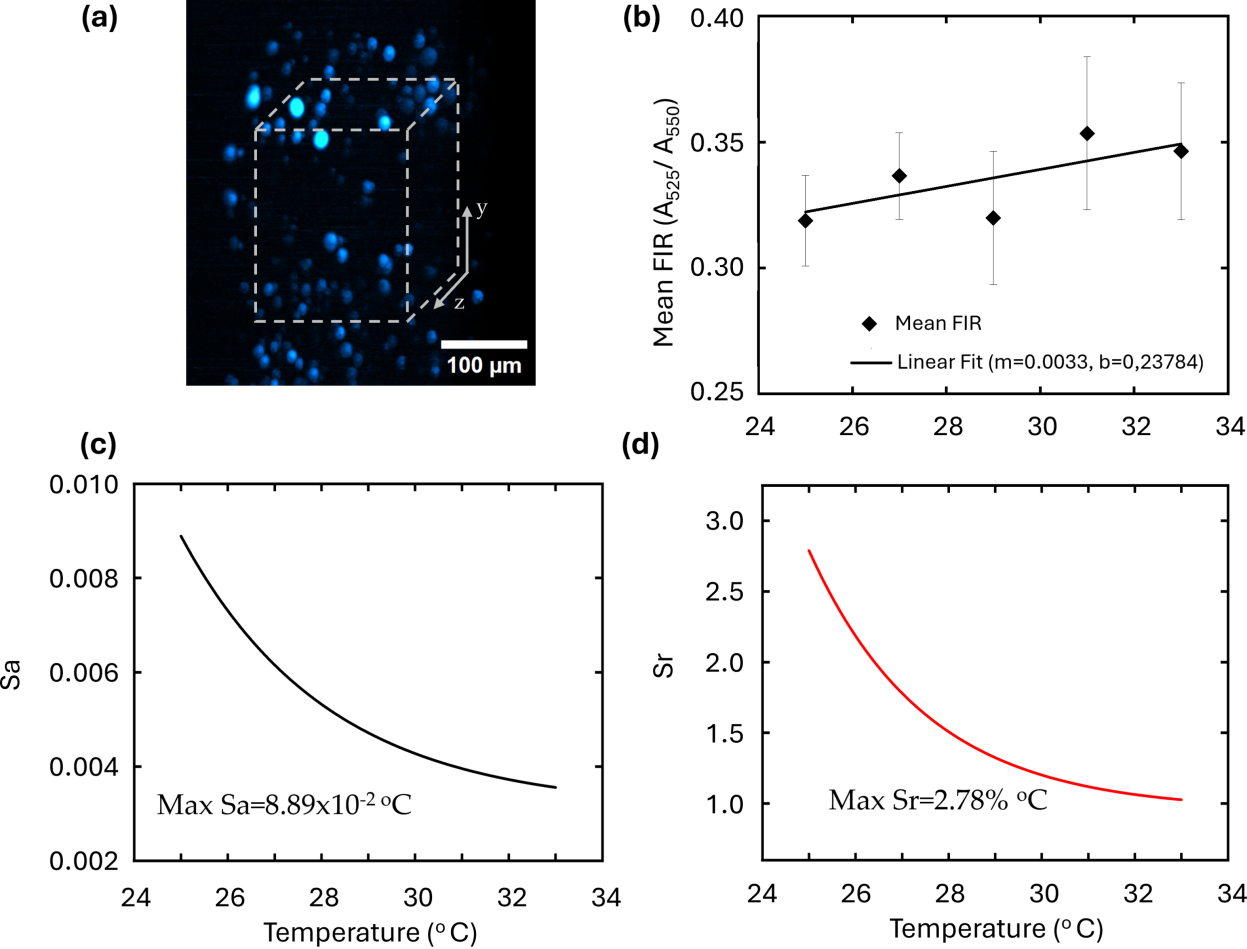
(a) Fluorescence intensity of UCNPs@lipids at 25 °C. (b) 3D temperature dependence from 25 to 33 °C. The black rhombs represent the mean values for *S*_1_, *S*_2_, and the combined selected nanoparticles. The solid line shows the linear fit of the calibration curve, obtained from the mean values of both UCNPs@lipids concentrations. (c) Absolute sensitivity (*S*_a_). (d) Relative sensitivity (*S*_r_). (a, b) where measured from 25 to 33 °C.

To accurately quantify temperature variations, a precise calibration curve must be established. Two UCNPs@lipids samples (S1) were prepared for FIR calibration, with concentrations of 0.5 and 2.5 mg·mL^−1^. Figure 4a presents the three-dimensional reconstruction of the UC fluorescence signal at 550 nm for the higher concentration (2.5 mg·mL^−1^) sample. The image reveals the characteristic aggregation of lipid-coated UCNPs, forming macroliposomes of approximately 30 µm diameter within the agarose gel. A corresponding volumetric image was also acquired at the 525 nm band (not shown) to compute the FIR. Pairs of these images were taken at different temperatures to establish the temperature-dependent FIR calibration. For each sample, approximately 25 macroliposomes were analyzed.

Although the FIR response follows a Boltzmann-type exponential temperature dependence (see Equation 1), our previous work demonstrated that within the 25–50 °C interval in NaYF_4_:Yb^3+^/Er^3+^systems, the FIR behavior of lipid-coated UCNPs can be well approximated by a linear dependence with a slope of *m* ≈ 0.003 °C^−1^ [40]. This finding is consistent with earlier experimental results showing a linear response up to 100 °C [44], confirming the robustness of this relationship across a broad thermal range. In the present study, the calibration was restricted to 25–33 °C to match the physiological temperature range of *C. elegans* and to evaluate the method’s sensitivity in detecting subtle temperature variations relevant to biological processes.

The resulting temperature-dependent FIR response is illustrated in Figure 4b, covering the 25–33 °C range, with measurements taken at 2 °C intervals. This temperature range was chosen because (i) it remains below the high-stress temperature threshold of *C. elegans* (>35 °C) [53] and (ii) it falls within the linear, high-sensitivity region of the NaYF_4_:Yb/Er nanoparticle thermometric response (20–100 °C) [44]. The black diamonds denote the combined mean values of both concentrations. A linear fit of the calibration curve (solid black line) was obtained for both UCNPs@lipids concentrations. The results closely align with Barron-Ortiz et al.’s previously reported calibration curve [40], showing a slope of *m* = 0.33 °C^−1^.

The resolution performance of the optical thermometer is assessed through its absolute (*S*_a_) and relative (*S*_r_) thermal sensitivities [35,54]. These are defined as:


[2] $$S_{\text{a}}=\left|\frac{\partial \text{FIR}}{\partial T}\right|,$$

[3] $$S_{\text{r}}=\frac{S_{\text{a}}}{\text{FIR}}\times 100\%.$$

The absolute sensitivity (*S*_a_) quantifies the FIR response to a 1 °C temperature change, and the relative sensitivity (*S*_r_) describes the FIR rate of change as a percentage of its value. Notice that the complex nature of the dynamic UC process, particularly at higher temperatures, can influence the temperature-sensing properties of UCNPs [37,42]. However, even within the physiological temperature range (30–50 °C), other UC processes such as thermal quenching, shifts in the Boltzmann distribution, and cross-relaxation, can also affect temperature sensing [37,54]. These effects may introduce variations in the FIR calibration, requiring careful evaluation for accurate thermometry [35,55]. This concern is particularly relevant in Er^3+^-based FIR thermometry as its sensitivity strongly depends on the energy gap between thermally coupled levels [42]. In our results, these effects are minimized, as the FIR calibration exhibits a consistent and linear temperature dependence over the 25–33 °C range, with no significant deviations from the expected response. This suggests that our UCNP system provides reliable temperature measurements, effectively mitigating potential distortions from UC artifacts. To demonstrate this, we calculated the thermal sensitivities *S*_a_ and *S*_r_ using Equation 2 and 3, based on the calibration obtained from the curve of Figure 4b. The calculated sensitivities are included in Figure 4c,d, showing that over the 25 to 33 °C temperature range, the absolute sensitivity *S*_a_ varied between 3.35 × 10^−3^ and 8.89 × 10^−3^ °C^−1^ (Figure 4c), while the relative sensitivity *S*_r_ ranged from 1.0 to 2.78%·°C^−1^ (Figure 4d). The largest values, *S*_a_ = 8.89 × 10^−3^ °C^−1^ and *S*_r_ = 2.78%·°C^−1^, were observed at 25 °C, with a decreasing trend as the temperature increased. It is important to note that the relative sensitivity remained above the 0.5%·C^−1^ threshold, a benchmark for high-performance nanothermometry applications [55]. This confirms previous studies showing that the temperature-dependent electronic 4S_3_/_2_→2H_11/2_ transitions in Er^3+^ provide optimal sensitivity within the 30–50 °C range, making them highly suitable for biological applications [42].

### *C. elegans* internal temperature measurement

After evaluating the 3D temperature sensing capability of UCNPs@lipids, we applied this technique to measure the internal temperature within fixed *C. elegans* nematodes. The worms were fed with the UCNPs@lipids solution at a 1 mg·mL^−1^ concentration, consistent with imaging and toxicity studies [56].

**Figure 5:**
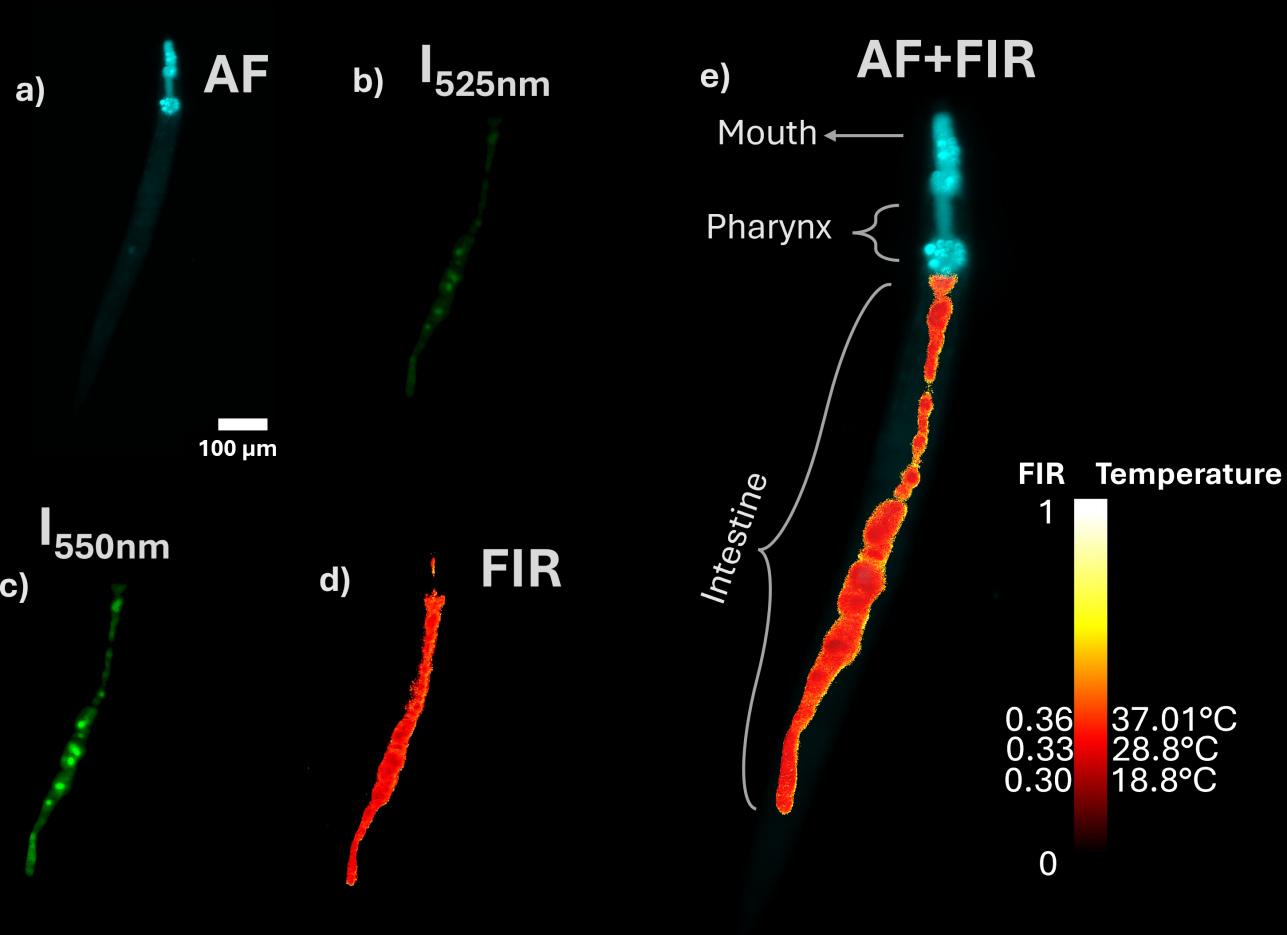
Temperature map inside of *C. elegans*. (a) Shows the autofluorescence in cyan, (b, c) Upconversion fluorescence images of the 525 and 550 nm emission bands at 25 °C. (d) FIR map. (e) The temperature map of *C. elegans* shows the FIR as a red-hot map and autofluorescence in cyan. The temperature signals are primarily localized within the intestinal lumen. Scale line: 100 μm. Temperature scale bar: to the left, FIR from 0 to 1, to the right, temperature, as a reference, 0.333 corresponds at approximately 28.8 °C.

Figure 5 illustrates the workflow for obtaining temperature maps. The process begins with identifying the nematode through its autofluorescence under 488 nm light-sheet excitation, highlighting key anatomical structures such as the pharynx and intestinal tract (Figure 5a). A *z*-stack is then acquired in steps of 2 μm to cover the whole volume of the nematode. Once the nematode is located, it is irradiated with a 980 nm NIR light sheet, and two emission filters (Thorlabs, MF510-40, and MF559-34) are used sequentially to collect the UC fluorescence bands centered at 525 and 550 nm. Figure 5b and Figure 5c show, respectively, the *z*-projections of the average fluorescence intensities for these bands. The FIR is then calculated plane-by plane, and the corresponding temperature map is generated using the calibration curve of Figure 4b. Figure 5e presents the *z*-axis projection of the FIR as a red-hot temperature map overlaid with the nematode’s autofluorescence (cyan), which delineates its morphology across the entire volume. The *C. elegans* intestine is a simple, tubular organ composed of 20 epithelial cells arranged into nine rings (int1–int9), spanning approximately 80% of the worm’s body length.

The lumen, located centrally within these rings, is lined with microvilli that facilitate nutrient uptake and particle retention [57]. Figure 5b–d shows the fluorescence emission throughout the intestinal structure post-ingestion, indicating UCNP accumulation within the lumen. This localization is strongly influenced by surface chemistry. The anionic character of our DOPS-rich lipid coating likely leads to electrostatic repulsion with the negatively charged glycocalyx of the intestinal cell membranes, thereby hindering endocytosis [58]. This contrasts with cationic coatings like polyethyleneimine (PEI), which promote endocytosis and lead to UCNP internalization into intestinal cells [59]. This observation is consistent with Chen et al. [50], who reported internalization of PEI-capped UCNPs into both the gut cavity and intestinal cells. They attributed UCNP uptake within the gut cavity to the favorable nanoparticle dispersibility and cellular internalization to endocytosis, facilitated by electrostatic interactions between the cationic PEI coating and negatively charged cell surfaces. The positive charge of PEI can be enhanced at lower pH levels, such as those found in parts of the *C. elegans* digestive tract [60], potentially promoting further endocytosis. In contrast, the UCNPs@lipids nanoparticles are negatively charged (due to abundant DOPS) and were observed in the intestine but not clearly within the intestinal cells. This suggests that endocytosis of these nanoparticles is sensitive to the specific capping ligand. Consequently, we attribute the recorded temperature distributions mainly to the luminal environment rather than intracellular regions, indicating that surface chemistry governs not only nanoparticle uptake but also the spatial interpretation of the thermal maps. This distinction is crucial, as it means our thermal maps report on the temperature within the digestive tract lumen, which may be influenced by ingested material and microbial activity, rather than the metabolic heat production of the intestinal cells themselves. To probe intracellular temperatures, a surface coating engineered for active cellular uptake would be required.

## Conclusion

We successfully demonstrated volumetric temperature measurements in *C. elegans* by combining LSFM with lipid-coated NaYF_4_:Yb^3+^/Er^3+^ upconversion nanoparticles. The lipid coating strategy proved crucial, enhancing biocompatibility and colloidal stability while enabling precise localization of the thermal probes within the nematode's digestive tract. Our calibration achieved excellent thermal sensitivity (up to 8.9 × 10^−3^ °C^−1^ absolute and 2.8%·°C^−1^ relative), well above the benchmark for high-performance nanothermometry.

While our experiments focused on fixed biological specimens, the 980 nm wavelength excitation and millisecond-scale slice acquisition inherent to LSFM make real-time thermometry in living organisms entirely feasible. This capability would enable monitoring metabolic or stress-induced temperature fluctuations at the single-organ level and evaluating thermal side effects of optogenetic, photothermal, or pharmacological treatments. Although our experiments used fixed *C. elegans*, the present study was conceived as a proof of concept to demonstrate the feasibility of volumetric FIR thermometry in biological systems using LSFM. In this context, PFA fixation is applied externally and primarily affects the nematode’s outer tissues. Aldehyde fixatives act at the organism’s surface without significantly altering the internal optical environment where the UC nanoparticles are located. Because upconversion luminescence arises from lanthanide ions shielded within the nanoparticle matrix, its emission is largely insensitive to minor dielectric or chemical variations in the surrounding medium [61]. Therefore, the overall influence of fixation on the recorded fluorescence and thermal response is negligible [62].

Extending this method to live imaging presents additional challenges related to the simultaneous acquisition of the thermally coupled emission bands. Sequential filter exchange limits the frame rate when using a single detector, whereas dual-camera configurations require precise spatial registration to avoid pixel mismatches and artifacts in the FIR calculation. To mitigate these limitations, we are currently developing an alternative approach based on color CMOS detection, which retrieves the spectral intensity from a single RGB image and eliminates the need for mechanical filter switching. This strategy, though beyond the scope of the present work, is expected to enable true real-time 3D nanothermometry in vivo. Future work will also focus on engineering UCNP coatings for specific subcellular targeting to further expand the biological applicability of this technique.

The readily transferable nature of both the lipid-coating approach and the optical setup establishes a practical foundation for extending this technique to other small model organisms, organoids, or individual mammalian cells. In summary, LSFM-enabled upconversion nanothermometry emerges as a powerful, non-invasive platform for probing spatiotemporal temperature dynamics across diverse biomedical applications, from cellular metabolism to organ-specific stress responses.

## Data Availability

Data generated and analyzed during this study is available from the corresponding author upon reasonable request.
